# Seasonal Variations in the Trace Elements and Mineral Profiles of the Bivalve Species, *Mytilus galloprovincialis*, *Chamelea gallina* and *Donax trunculus*, and Human Health Risk Assessment

**DOI:** 10.3390/toxics11040319

**Published:** 2023-03-28

**Authors:** Katya Peycheva, Veselina Panayotova, Rositsa Stancheva, Albena Merdzhanova, Diana Dobreva, Vincenzo Parrino, Nicola Cicero, Francesco Fazio, Patrizia Licata

**Affiliations:** 1 Department of Chemistry, Medical University of Varna, 9002 Varna, Bulgaria; 2 Department of Chemical, Biological, Pharmaceutical and Environmental Sciences, University of Messina, 98168 Messina, Italy; 3 Department of Biomedical and Dental Sciences and Morphofunctional Imaging, University of Messina, 98168 Messina, Italy; 4 Department of Veterinary Sciences, University of Messina, Polo Universitario Annunziata, Via Palatucci 13, 98168 Messina, Italy

**Keywords:** heavy metals, bivalves, target hazard quotient, hazard index, target risk

## Abstract

This study aimed to provide data on selected toxic (Cd, Pb and Ni), essential (Cr, Cu, Fe, Mn and Zn) and microelement (Na, K, Ca and Mg) concentrations in edible tissues of the Mediterranean mussel (*Mytilus galloprovincialis*), striped venus clam (*Chamelea gallina*) and the wedge clam (*Donax trunculus*). Samples were collected from the Black Sea (Bulgaria) four times over, a period of one year (2022). In comparison with the maximum permissible levels set by the EU and USFDA, all elemental concentration found in the bivalve species were lower than the prescribed limits. An estimation of the dietary metal intake through calculation of the target hazard quotients (THQ), hazard index (HI) and target risk (TR) was performed. The target hazard quotient (THQ) for individual metal and HI for combined metals were lower than 1, indicating no health risk for consumers due to the intake of either individual element or combined ones. The target risk value for toxic inorganic Pb and Cr was below 10^−6^, indicating no carcinogenic risk. According to these results, the consumption of these bivalve species is completely safe for human health.

## 1. Introduction

Over the past few decades, the consumption of shellfish has increased due to the potential beneficial properties [1,2,3,4,5]. Shellfish are excellent sources of proteins, fatty acids, vitamins (niacin, thiamine) and minerals (Ca, Mg, Fe, and Zn) [4]. Despite the numerous human health effects, various contaminants, such as heavy metals, could pose a risk to regular consumers [6]. All metals possess a negative impact on human health when their intake exceeds certain levels, but lead, cadmium and nickel are very toxic, even at low doses [7]. Other elements (such as Cu, Fe, Zn and Se) are essential for humans, but they may become toxic at high concentrations, as well [7,8]. This is the reason why it is of great importance to determine marine organisms’ chemical composition, regarding the content of heavy metal, in order to evaluate the possible risks to human health [8].

Among mollusks, the Mediterranean mussel (*Mytilus galloprovincialis)*, striped venus clam (*Chamelea gallina*) and the wedge clam (*Donax trunculus*) are the most harvested and cultivated species in the world [1,2]. *M. galloprovincialis* is widely distributed in the coastal waters of the eastern Atlantic–Mediterranean region and is mainly farmed in the northwest coastal waters of Spain, Italy, Turkey and Bulgaria [9,10]. *C. gallina* is distributed throughout the entire Mediterranean, Black and Marmara Sea coasts, and is a filter-feeder, muddy sand species [1]. Black Sea *C. gallina* prefers greater depths of 5 to 18 m (rarely up to 25+ m) [11]. The benthic *D. trunculus* is an Atlantic–Mediterranean warm-temperate species distributed from Senegal to the northern Atlantic coast of France, the Mediterranean and the Black Sea, as well as along the Marmara Sea [11,12,13]. In the Bulgarian Black Sea, *D. trunculus* dominates the wave action upper infralittoral medium and fine sands, usually between 1 and 6.5 m (but is also observed from 0.9 to 9 m) [11].

The Black Sea is an enclosed sea isolated from the World Ocean and the large inflows of river water distinguish it from other sea basins [14]. One of the most polluted seas with higher degree of eutrophication in recent years in the world is the Black Sea [15]. The pollution of the Black Sea increases each year, which leads to a decline in fishery yield and has a serious impact on the tourist industry. The Mediterranean mussel (*Mytilus galloprovincialis*), striped venus clam (*Chamelea gallina*) and the wedge clam (*Donax trunculus*) are bivalve species of huge economic importance in the Black Sea region [15].

The aim of this study was to determine the seasonal dynamics of trace (Cd, Cr, Cu, Fe, Mn, Ni, Pb, Zn) and macro element (Ca, K, Mg, Na) content of three Black Sea bivalve species, *Donax trunculus*, *Mytilus galloprovincialis* and *Chamelea gallina*, over a period of one year, and to calculate the target hazard quotients (THQ), hazard index (HI) and target risk (TR) values for Pb and Cr as developed by the Environmental Protection Agency (EPA) in the US for the estimation of potential health risks associated with long-term exposure to chemical pollutants [16].

## 2. Materials and Methods

### 2.1. Sampling and Sample Treatment

The study was conducted four times during a one-year period (2022) on wild populations of the bivalves. The samples were collected from the area of Azalia resort zone at two points (43.24642° N 28.01972° E; 43.24781° N 28.02058° E), and from the area of Krapec to the resort St. Konstantin and Elena at four points (43.587° N 28.609° E; 43.434° N 28.37° E; 43.246° N 28.02° E; 43.247° N 28.022° E). The *Donax trunculus* abundance was assessed, based on bottom trawling with a beam trawl in 1–3 m isobaths, while *Mytilus galloprovincialis* and *Chamelea gallina* within 1–8 m isobaths. Only clams with undamaged valves were studied. More than 2 kg of bivalves with comparable shell lengths were collected, placed into plastic bags and brought to the laboratory. The bivalves were cleaned, rinsed with Milli-Q water, brushed, shucked and soft tissues were removed with a Teflon knife and stored in polyethylene bags at −20 °C until analysis. Around two hundred specimens of each species were taken randomly for the determination of sample mean. The physical parameters of sampled shellfish were as follows (Table 1):

### 2.2. Reagents and Standard Solutions

All solutions were prepared with analytical reagent grade chemicals and ultra-pure water (18 MΩ cm) generated by purified distilled water with a Millipore Milli-Q Gradient A-10 water purification system (Bedford, MA, USA). All the plastic and glassware were cleaned by soaking in 2 M HNO_3_ for 48 h, and rinsed five times with distilled water, and then five times with deionized water prior to use. The calibration standard solutions were freshly prepared by dilution of Optima Family Multi-Element Standard, Matrix per Volume: 2% HNO_3_, and Multi-Element Calibration Standard 3, Matrix per Volume: 5% HNO_3_ stock solution (Perkin Elmer^®^, Waltham, MA, USA).

### 2.3. Analytical Procedure

Wet digestions were performed in triplicate by weighing approximately 1.0 g of the tissues with a mixture of 8 mL HNO_3_ (65% Merck, Suprapur, Darmstadt, Germany) and 2 mL H_2_O_2_ (30–32% Optima^TM^, for Ultra Trace Analysis, Fisher Chemical, Waltham, MA, USA) in a microwave closed-vessel digestion system MARS 6 (CEM Corporation, Matthews, NC, USA). The power system used provides continues microwave emission at each level (Table 2). After digestion the samples were diluted to 25 mL in acid-washed polyethylene bottles with Milli-Q water, and stored prior to analysis. A blank digest was performed in the same way. The concentrations of Ca, K, Mg, Na, Cd, Cr, Cu, Fe, Mn, Ni, Pb, Zn in the samples were determined using ICP-OES Spectrometer (Optima 8000, Perkin Elmer, Waltham, MA, USA) with the following operating conditions: plasma gas flow—8 L/min; auxiliary gas flow—0.4 L/min; nebulizer gas flow—0.6 L/min; RF power—1500 watts; plasma view—axial/radial; nebulizer—concentric glass, MEINHARD^®^ Type C.

### 2.4. Quality Assurance and Quality Control of the Method

Five sub-samples of each material were digested along with the blank sample, using the methods described above. All measurements were performed in triplicate for the samples and standard solutions. Metal contents were calculated and shown as mg/kg of wet weight. The accuracy of the procedure for the determination of trace metals in mollusks was tested using ERM-CE 278 k (mussel tissues from European Commission, joint research center, Belgium) certified reference material. The CRM was digested and analyzed in the same way as the analytical samples. The recovery values were between 86 and 103% for the individual elements.

### 2.5. Hazard Risk Estimation

#### 2.5.1. Target Hazard Quotient (THQ)

The THQ, developed by USEPA [16], is used to assess the human health risk for non-carcinogenic elements in the local human population over a lifetime, in comparison with the reference oral dose (RfD) [17]. THQ was calculated as per USEPA Region III Risk Based Concentration Table [18], by using the following equation:

$$THQ=\frac{\left(M_{C}\cdot I_{R}\cdot 10^{-3}\cdot EF\cdot ED\right)}{RfD\cdot BW_{a}\cdot AT_{n}}$$

where *M_C_* is the heavy metal concentration in bivalve species (mg/kg ww), *I_R_* is the average daily consumption of shellfish (0.8 g/person/day) [19], *EF* is the exposure frequency (365 days/year), *ED* is the exposure duration (30 years or 10,950 days) for non-cancer risk as used by USEPA [18], *RfD* is the reference dose of individual metal (0.001 μg/g day for Cd, 0.04 μg/g day for Cu, 0.003 μg/g day for Cr, 0.009 μg/g day for Fe, 0.02 μg/g day for Ni, 0.004 mg/kg for Pb, 0.14 mg/kg for Mn and 0.3 μg/g day for Zn) [18], *BW_a_* is an average adult body weight (70 kg average) and *AT_n_* is the average exposure time for non-carcinogens [18]. Values of *THQ* below 1 show no harmful effect for human health. 

#### 2.5.2. Hazard Index (HI)

The hazard index from *THQ*s is expressed as the total of the hazard quotients [20]:

$$HI=THQ_{Cd}+THQ_{Cu}+THQ_{Cr}+THQ_{Fe}+THQ_{Ni}+THQ_{Pb}+THQ_{Mn}+THQ_{Zn}$$

#### 2.5.3. Target Risk (TR)

Target cancer risk (*TR*) indicates carcinogenic risks. The model for estimating TR is shown as follows:

$$TR=\frac{C\cdot C_{R}\cdot 10^{-3}CPS_{o}\cdot EF\cdot ED}{BW_{a}\cdot AT_{c}}$$

where *C* is the metal concentration in the mollusk species (mg/kg ww), *C_R_* is the average daily consumption of shellfish (g/day) (0.8 g/person/day) [19], *CPS_o_* is the carcinogenic potency slope, oral (Cr = 0.5 mg/kg bw-day, Pb = 0.0085 mg/kg bw-day), *AT_c_* is the averaging time, carcinogens (day/years), and was calculated by multiplying exposure frequency in exposure duration over lifetime. The *TR* value for intake of Cr, Ni and Pb was calculated to indicate the carcinogenic risk, since Cd, Cu, Fe, Mn and Zn do not cause any carcinogenic hazards according to the USEPA. The acceptable values of *TR* are 1 × 10^−6^ according to the USEPA [18].

### 2.6. Statistical Analysis

All analyses were performed in 5 replicates and the results were expressed as the mean values ± standard deviation (SD). The mean values of trace elements were compared by a one-way ANOVA, followed by a post hoc Tukey’s test. Statistical significance was considered at *p* ≤ 0.05 (GraphPad Prism 5).

## 3. Results and Discussion

### 3.1. Trace Elements in Bivalve Species Tissues

Mean concentrations and standard deviations of toxic and essential elements of the three bivalve species from the region of the Black Sea Bulgaria are shown in Table 3. The summarized results of this study are expressed as means (mg/kg) of wet weight (ww).

In this study, significant differences in elemental content were recorded between different seasons (Table 3), especially between winter and summer samples. An increase in the concentration of some elements during cold months corresponds to the period of the high storage of energy reserves, before the reproduction period, as many authors reported [21,22].

Cd is a non-essential element for the human body and it is capable of producing chronic toxicity, present at minimal concentration of 1 mg/kg [22]. The concentration of Cd ranged between 0.22 mg/kg ww and 0.91 mg/kg ww for *Chamelea gallina*; between 0.28 mg/kg ww and 0.74 for *Mytilus galloprovincialis*; between 0.03 mg/kg ww and 0.09 for *Donax trunculus*. The maximum Cd level permitted for bivalve mollusks intended for human consumption is 1.0 mg/kg ww according to the European Community and Bulgarian Food Regulation [23]. In the literature, cadmium concentration has been reported to range from 0.204 to 0.426 mg/kg ww for *C. gallina* [1], 0.024 to 0.159 mg/kg ww for *D. trunculus* [1], 0.292 to 0.970 mg/kg ww for *M. galloprovincialis* [2], all sampled from the Sea of Marmara, Turkey; between 0.17 and 0.64 mg/kg ww in wild and farmed *M. galloprovincialis* from Boka Kotorska Bay, south east Adriatic Sea [9]; between 0.34 and 1.71 mg/kg dw in whole soft tissue of mussels collected at 14 locations along the eastern Adriatic coast, Croatia [24]. Cadmium levels in the present study were in good agreement with the reported literature data and data from international organizations.

The lead concentration of *C. gallina* ranged from 0.02 mg/kg ww to 0.55 mg/kg ww; between 0.07 and 0.41 mg/kg ww for *M. galloprovincialis*; between 0.05 and 0.17 mg/kg ww for *Donax trunculus*. The maximum Pb concentration for all species was found in the winter season. According to the European Commission Regulation (1881/2006/EC) [23], the maximum acceptable concentration (MAC) of Pb in bivalve mollusks is 1.5 mg/kg ww. Őzden et al. (2009) reported values of Pb up to 1.342 mg/kg ww for *C. gallina* [1], up to 0.916 mg/kg ww for *D. trunculus* [1], and between 0.292 and 0.970 mg/kg ww for *M. galloprovincialis* [2] from the Sea of Marmara. In their study of mussel tissues in the Eastern Black Sea, Turkey, Čevik et al. [25] found Pb levels between 5.9 and 45.9 μg/g. As a result of the Pearson correlation in the study, metal concentration in the tissues did not seem to depend on the mussel length and sampling site, according to the same authors [25]. Cohen et al. [26] reported values of 0.80 μg/g dw for the *M. galloprovincialis* species in three southern California coastal wetlands. Zhelyazkov et al. [6] declared the mean Pb levels of 0.251 ± 0.141 for wild *M. galloprovincialis* from Varna Bay, Bulgaria. The results from the current study are within the MAC and the data in the literature.

Nickel normally occurs at very low levels in the environment and seafood is one of the major exposure paths. High amounts of Ni may lead to skin allergies, chronic bronchitis, reduced lung function, and cancer of the lung and nasal sinus. The minimum concentration was found in *D. trunculus* (0.005–0.006 mg/kg ww), while the other two species are characterized with higher concentrations (0.003–0.51 mg/kg ww for *C. gallina* and 0.04–0.21 mg/kg ww for *M. galloprovincialis*). The maximum Ni level permitted for muscle meat of some marine fish is 0.5 mg/kg ww according to the Bulgarian Food Regulation [27]. Nickel concentration in the literature has been reported to range from 0.13 to 0.43 mg/kg ww in *M. galloprovincialis* from the Italian coast, West Adriatic Sea [28]; 67.8 to 1948 ng/g ww in blue mussels (*M. edulis* Complex) from the Baltic Sea [29]; 2194 to 6180 μg/kg dw in mussel samples from Durres and Vlora “hot spots”, Albania [30]; 0.673 to 1.618 mg/kg ww in *C. gallina* and 1.097 and 7.505 mg/kg ww in *D. trunculus* [1]. The concentrations of Ni in our study were below that reported in the literature and by various health organizations.

Cr, Cu, Fe, Mn and Zn are essential elements for humans and most of them occur naturally in many food sources. The content of Zn was different between species. The maximum Zn concentration was observed in the winter season for all species, following the order: *D. trunculus* (27.46 mg/kg ww) > *M. galloprovincialis* (21.41 mg/kg ww) > *C. gallina* (19.75 mg/kg ww). There was no difference among the species in the summer period. Őzden et al. (2009) concluded that the concentrations of Zn were significantly higher in *D. trunculus* than in *C. gallina* (*p* < 0.05) [1], similarly to other authors [4,31,32,33]. Concerning the Cu concentration of the species under analyses, it is with maximum value during the winter season (*D. trunculus* (26.53 ± 1.71 mg/kg); *C. gallina* (9.27 ± 0.05 mg/kg); *M. galloprovincialis* (2.72 ± 0.30 mg/kg)). These results are higher than the ones found by Őzden et al. [1,2] and are in the range determined for *M. galloprovincialis* by other authors [24,25,32]. The Fe levels in bivalve species ranged from 13.78 mg/kg to 120.57 mg/kg for *D. trunculus*. There is no maximum permitted level for Fe in bivalve species according to European legislation [23], but the US Food and Drug Administration set 80 mg/kg ww as the limit [20]. The iron concentration found in the literature was in agreement with the data from this study [1,2,9,28]. Mn values in the literature vary between 41 and 59 μg/g ww in tissues of mussels from the Eastern Black Sea [25]; 0.865 and 11.306 mg/kg ww in wild *M. galloprovincialis* from Bosporus of the Sea of Marmara [2]; 1.33 and 3.85 mg/kg w/w found in *M. galloprovincialis* at three locations in Boka Kotorska during four different seasons in 2015 [7]. There are no data regarding the maximum Mn level permitted according to the Bulgarian Food Codex [27], but the data from current study are clearly within those found in the literature.

### 3.2. Macro Elements

The macro elements studied were K, Na, Ca, and Mg (Table 4). Of all the elements, Na, K and Ca were the most abundant. The concentration range for these elements in the studied species was, in general, within the range reported by several authors [2,25,34]. Usually, seafood has lower contents of Na when compared to K, both in fish or bivalves from salt or fresh water [35].

Of all species, *C. gallina* displayed the highest levels of Mg (958.52 ± 12.17 mg/kg ww) during the autumn season, but still less than reported by Nekhoroshkov et al. [34] (1020–4320 μg/g ww for *M. galloprovincialis* along the South African coastline), and within the values reported by Őzden et al. [2]. The minimum concentration of this element has been found for *Mytilus galloprovincialis* (341.51 mg/kw).

Further, in winter, *Mytilus galloprovincialis* had the highest Ca, K, Mg and Na concentration. Such an increasing trend across the four seasons was not observed for the other species subject to this study.

### 3.3. Nutritional Contribution of the Species in Terms of Essential Elements (Benefits)

The nutritional contribution (in DRI percent) of each essential element was calculated based on Dietary Reference Intakes (DRI) [36]. The estimated daily intake (EDI) for each element was calculated using the element average concentration (mg/kg) obtained for each mollusk, considering a meal of 150 g of bivalve [35].

Conserning Ca, *D. trunculus* is the bivalve that can provide the highest proportion (48.8% of the DRI) of the daily required intake of this element. *C. gallina* can contribute to the highest percent of Mg (34.2% of the DRI), while *M. galloprovincialis* contributes most to the DRI of Na (38.1% of the DRI) and K (10.7% of the DRI).

### 3.4. Potential Health Risk Assessment

The target hazard quotients for Cd, Cr, Cu, Fe, Mn, Ni, Pb and Zn hazard indexes and target risks for Cr and Pb, estimated through the consumption of the three bivalve species, are shown in Table 5.

THQ is an integrated risk index that compares the ingested amount of a contaminant with a standard reference dose [34]. The health risk assessment is done based on assumptions. The acceptable value for THQ equals to 1, according to the USEPA [18]. The elements Cd, Cr, Cu, Fe, Mn, Ni, Pb and Zn did not exceed 0.1 of THQ, and the THQ was less than 1 for all elements. Perošević et al. found that the THQ_Fe_ was between 0.014 and 0.044, THQ_Zn_ 0.015–0.050, THQ_Mn_ 0.003–0.011, THQ_Cu_ 0.007–0.019, THQ_Ni_ 0.004–0.024, THQ_Pb_ 0.224–0.297, THQ_Cr_ 0.017–0.071 and THQ_Cd_ 0.051–0.187 for mussels *M. galloprovincialis* from Boka Kotorska Bay, Montenegro [7], and THQs in the range of 0.3–0.7 for the elements Cr, Co, Zn, and I for wild mussels along the South African coastline [34].

The HI values, which evaluate an additive risk from different elements, were lower than 1 for each bivalve sample, which indicates that there is no concern for potential health effects.

The Environmental Protection Agency (EPA) defines TR as “the incremental probability of an individual to develop cancer over a lifetime, as a result of exposure to a potential carcinogen” [17]. The cancer slope factor (CSF) value (μg kg^−1^ day^−1^), according to USEPA, is only available for As, Pb and Cr [17]. A risk level of 1 × 10^−6^ has been considered the point of excess cancer risk, indicating 1 per 1,000,000 chance of getting cancer via consumption of shellfish toxic metals, estimated for 70 years [37]. The safe point for carcinogenic risks must be lower than this level [37]. The range of risks set by the EPA is 1 × 10^−4^ to 1 × 10^−6^, and it is deemed unacceptable if the risks are surpassing 1 × 10^−4^. A carcinogenic risk of 1 × 10^−4^ poses health hazards; therefore, if sufficiently large, health hazards need some sort of intervention and remediation [8]. The calculated values in this study are within the range of 10^−6^, suggesting that the intake of Cr and Pb by consumption of these bivalve species would not result in appreciable hazard risk of the human body. As a conclusion, the levels of Cd, Cr, Cu, Fe, Mn, Ni, Pb and Zn in mussels could be considered relatively safe for consumption according to the hazardous quotients and indexes.

## 4. Conclusions

The toxic, essential and macro element concentrations in Mediterranean mussel (*Mytilus galloprovincialis*), striped venus clam (*Chamelea gallina*) and the wedge clam (*Donax trunculus*) from Bulgarian coast of the Black Sea, at different seasons, did not exceed the European Union and US Food and Drug Administration requirements, and are within the maximum acceptable limits set by those health organizations. In terms of the THQ and HI values of the elements, the continuous consumption of these shellfish species in this area may not create health risk over a prolonged period. The target risk value for Cr and Pb was below 10^−6^, indicating no carcinogenic risk. Based on the results from this study, the consumption of these bivalve species is safe for human health.

## Figures and Tables

**Table 1:** Physical parameters of sampled shellfish (mean ± standard deviation).

	Spring	Summer	Autumn	Winter
Striped venus clam (*C. gallina*)				
Total weight (g)	3.01 ± 0.12	3.15 ± 0.09	3.09 ± 0.02	3.32 ± 0.39
Soft tissue weight (g)	1.42 ± 0.68	1.46 ± 0.22	1.51 ± 0.26	1.54 ± 0.32
Total size (cm)	2.09 ± 0.34	2.11 ± 0.14	2.01 ± 0.84	2.31 ± 0.61
Mussel (*M. galloprovincialis*)				
Total weight (g)	3.00 ± 0.59	3.06 ± 0.51	3.60 ± 0.29	3.90 ± 0.69
Soft tissue weight (g)	1.89 ± 0.12	1.76 ± 0.34	1.87 ± 0.05	1.95 ± 0.13
Total size (cm)	4.41 ± 1.52	4.61 ± 0.31	5.10 ± 0.9	4.20 ± 1.12
Wedge clam (*D. trunculus*)				
Total weight (g)	4.02 ± 0.59	4.10 ± 0.49	4.32 ± 0.29	4.91 ± 0.05
Soft tissue weight (g)	1.56 ± 0.12	1.13 ± 0.03	0.89 ± 0.13	1.26 ± 0.13
Total size (cm)	2.9 ± 1.52	3.1 ± 0.19	3.3 ± 0.92	3.4 ± 1.11

**Table 2:** Microwave digestion system general and operational parameters.

Microwave Digestion System “MARS 6”, Acid Mixture		Step	Initial Power (W)	Time (min)	Final Power (W)	Fan
T °C (max)	200 °C	1	100	5	600	1
Pressure (max)	75 bar	2	600	5	600	1
Quartz vessels	EasyPrep^TM^ Plus	3	600	5	800	1
Sample amount	1 g	4	800	15	800	1

**Table 3:** Toxic and essential content (mg/kg ww) of the bivalve species (Black Sea, Bulgaria).

	Toxic Elements			Essential Elements				
	Cd	Pb	Ni	Zn	Cr	Cu	Fe	Mn
*Chamelea gallina*								
Spring	0.24 ± 0.01 ^c^	0.03 ± 0.01 ^c^	0.03 ± 0.01 ^b^	8.68 ± 0.31 ^c^	0.05 ± 0.03 ^b^	1.34 ± 0.06 ^c^	30.35 ± 2.83 ^b^	0.69 ± 0.03 ^b^
Summer	0.43 ± 0.08 ^b^	0.11 ± 0.02 ^b^	0.11 ± 0.09 ^b^	13.96 ± 1.47 ^b^	0.17 ± 0.05 ^b^	1.89 ± 0.35 ^b^	53.19 ± 18.27 ^a^	1.42 ± 0.32 ^a^
Autumn	0.22 ± 0.05 ^c^	0.12 ± 0.02 ^b^	0.13 ± 0.13 ^b^	7.96 ± 3.41 ^c^	0.07 ± 0.03 ^b^	1.73 ± 0.51 ^b^	16.62 ± 5.12 ^c^	1.57 ± 0.19 ^a^
Winter	0.91 ± 0.02 ^a^	0.55 ± 0.03 ^a^	0.51 ± 0.41 ^a^	19.75 ± 0.38 ^a^	1.53 ± 0.13 ^a^	9.27 ± 0.34 ^a^	48.76 ± 5.51 ^a^	0.71 ± 0.02 ^b^
*Mytilus galloprovincialis*								
Spring	0.28 ± 0.03 ^c^	0.07 ± 0.02 ^b^	0.04 ± 0.01 ^b^	16.00 ± 3.75 ^b,c^	0.01 ± 0.01 ^d^	0.38 ± 0.05 ^d^	13.98 ± 1.11 ^c^	0.83 ± 0.04 ^b^
Summer	0.44 ± 0.07 ^b^	0.11 ± 0.02 ^b^	0.15 ± 0.09 ^a,b^	14.21 ± 1.38 ^c^	0.17 ± 0.04 ^c^	1.92 ± 0.32 ^b^	53.91 ± 1.72 ^b^	1.44 ± 0.30 ^b^
Autumn	0.30 ± 0.03 ^b,c^	0.11 ± 0.01 ^a,b^	0.21 ± 0.10 ^a^	24.97 ± 6.03 ^a^	0.28 ± 0.06 ^b^	0.90 ± 0.17 ^c^	108.07 ± 7.33 ^a^	3.27 ± 0.75 ^a^
Winter	0.74 ± 0.06 ^a^	0.41 ± 0.13 ^a^	0.13 ± 0.03 ^a,b^	21.41 ± 1.52 ^a,b^	0.36 ± 0.05 ^a^	2.72 ± 0.30 ^a^	59.16 ± 3.54 ^b^	0.97 ± 0.01 ^b^
*Donax trunculus*								
Spring	0.04 ± 0.01 ^a,b^	nd	nd	10.85 ± 1.07 ^b^	nd	11.34 ± 2.71 ^c^	13.92 ± 1.78 ^d^	0.68 ± 0.06 ^a^
Summer	0.04 ± 0.01 ^a,b^	0.05 ± 0.01 ^b^	nd	12.47 ± 0.79 ^b^	0.11 ± 0.01 ^a^	16.01 ± 1.29 ^b^	39.78 ± 4.24 ^c^	1.98 ± 0.73 ^a^
Autumn	0.03 ± 0.02 ^b^	0.05 ± 0.03 ^b^	0.005 ± 0.008 ^a^	7.91 ± 5.18 ^b^	0.36 ± 0.43 ^a^	14.13 ± 2.88 ^b,c^	83.24 ± 1.63 ^b^	2.47 ± 1.72 ^a^
Winter	0.09 ± 0.05 ^a^	0.17 ± 0.09 ^a^	0.006 ± 0.131 ^a^	27.46 ± 2.53 ^a^	0.44 ± 0.10 ^a^	26.53 ± 1.71 ^a^	120.57 ± 13.52 ^a^	0.37 ± 0.04 ^b^

Legend: mean values ± standard deviation; values in a column not sharing a common superscript are significantly different between seasons (*p* < 0.05); nd—not detected.

**Table 4:** Concentration (mean ± standard deviation) and nutritional contribution of 150 g serving portion for *Chamelea gallina*, *Mytilus galloprovincialis* and *Donax trunculus*, in terms of essential elements.

	DRI /mg/day/		*Chamelea gallina*			*Mytilus galloprovincialis*			*Donax trunculus*		
			mg/kg	EDI	%DRI	mg/kg	EDI	%DRI	mg/kg	EDI	%DRI
Ca	1000–**1200** *	Sp	786.39 ± 91.04	118	9.8	1365.64 ± 30.16	205	17.1	1167.42 ± 37.18	175	14.6
		S	749.50 ± 96.75	112	9.4	588.27 ± 32.43	88	7.4	806.05 ± 37.53	121	10.1
		A	1759.14 ± 46.52	264	22.0	385.48 ± 37.08	58	4.8	1946.55 ± 37.08	292	24.3
		W	1173.00 ± 51.31	176	14.7	2282.43 ± 38.76	342	28.5	3905.96 ± 26.82	586	48.8
K	**4700** *	Sp	1605.20 ± 66.03	241	5.1	2505.04 ± 19.54	376	8.0	2559.81 ± 58.16	384	8.2
		S	1527.33 ± 37.15	229	4.9	1581.99 ± 52.03	237	5.0	2820.50 ± 13.09	423	9.0
		A	2171.43 ± 29.29	326	6.9	1701.83 ± 66.07	255	5.4	1171.04 ± 17.67	176	3.7
		W	1891.00 ± 15.39	284	6.0	3346.48 ± 19.23	502	10.7	1702.34 ± 13.02	255	5.4
Mg	310–**420** **	Sp	573.52 ± 31.95	86	20.5	341.51 ± 10.93	51	12.2	511.67 ± 12.30	77	18.3
		S	546.22 ± 36.59	82	19.5	388.29 ± 13.58	58	13.9	471.26 ± 13.77	71	16.8
		A	958.52 ± 12.17	144	34.2	426.98 ± 17.42	64	15.2	493.85 ± 12.95	74	17.6
		W	452.95 ± 14.73	68	16.2	773.45 ± 21.73	116	27.6	742.57 ± 15.13	111	26.5
Na	1200–**1500** *	Sp	2089.37 ± 86.07	313	20.9	1835.89 ± 7.33	275	18.4	2223.96 ± 13.06	334	22.2
		S	1988.43 ± 66.81	298	19.9	1150.27 ± 64.09	173	11.5	2112.19 ± 14.63	317	21.1
		A	3804.41 ± 16.36	571	38.0	2019.48 ± 47.27	303	20.2	2668.44 ± 29.77	400	26.7
		W	2180.00 ± 0.96	327	21.8	3813.63 ± 18.36	572	38.1	2699.63 ± 14.76	405	27.0

Legend: Sp—spring; S—summer; A—autumn; W—winter; DRI (in bold)—recommended daily intake values [36], used in the calculation of % DRI; EDI—estimated daily intake; * AI—adequate intake; ** RDA—recommended dietary allowances.

**Table 5:** Risk values (*THQ*, *HI* and *TR*) of each metal contaminant in bivalve species, Black Sea (Bulgaria).

	Target Hazard Quotient (THQ)								Hazard Index (HI)	Target Risk (TR)	
	Cd	Cr	Cu	Fe	Mn	Ni	Pb	Zn		Cr	Pb
*Chamelea gallina*											
Spring	0.0027	0.0002	0.0004	0.0005	0.0001	0.00002	0.0001	0.0003	0.004	1.2 × 10^−7^	1.2 × 10^−9^
Summer	0.0049	0.0007	0.0005	0.0009	0.0001	0.00009	0.00003	0.0005	0.008	4.2 × 10^−7^	4.4 × 10^−9^
Autumn	0.0025	0.0003	0.0005	0.0003	0.0001	0.00007	0.0001	0.0003	0.004	1.8 × 10^−7^	7.7 × 10^−10^
Winter	0.0129	0.0058	0.0026	0.0008	0.0001	0.00029	0.00018	0.0008	0.025	3.7 × 10^−6^	2.3 × 10^−8^
*Mytilus galloprovincialis*											
Spring	0.0032	0.0001	0.0001	0.0002	0.0001	0.00002	0.0002	0.0006	0.004	3.5 × 10^−8^	2.9 × 10^−9^
Summer	0.0050	0.0007	0.0005	0.0009	0.0001	0.00009	0.00004	0.0005	0.008	4.3 × 10^−7^	4.7 × 10^−9^
Autumn	0.0035	0.0011	0.0003	0.0018	0.0003	0.00012	0.0001	0.0010	0.008	6.8 × 10^−7^	7.8 × 10^−10^
Winter	0.0084	0.0014	0.0008	0.0010	0.0001	0.00007	0.0013	0.0008	0.014	8.9 × 10^−7^	1.7 × 10^−8^
*Donax trunculus*											
Spring	0.0004	-	0.0032	0.0002	0.00006	-	-	0.0004	0.004	-	-
Summer	0.0005	0.0004	0.0017	0.0006	0.00016	-	0.0002	0.0005	0.004	2.7 × 10^−7^	2.2 × 10^−9^
Autumn	0.0003	0.0014	0.0012	0.0014	0.00020	0.000003	0.0002	0.0003	0.005	8.7 × 10^−7^	2.0 × 10^−9^
Winter	0.0010	0.0017	0.0076	0.0020	0.00003	0.000003	0.0006	0.0010	0.014	1.1 × 10^−6^	7.4 × 10^−9^

## Data Availability

Not applicable.
